# Role of T_H_17 Responses in Increasing Herpetic Keratitis in the Eyes of Mice Infected with HSV-1

**DOI:** 10.1167/iovs.61.6.20

**Published:** 2020-06-09

**Authors:** Satoshi Hirose, Ujjaldeep Jaggi, Shaohui Wang, Kati Tormanen, Yoshiko Nagaoka, Makoto Katsumata, Homayon Ghiasi

**Affiliations:** 1 Center for Neurobiology and Vaccine Development, Ophthalmology Research, Department of Surgery, Cedars-Sinai Medical Center, Los Angeles, California, United States; 2 Mouse Genetic Core, Department of Biomedical Science, Cedars-Sinai Medical Center, Los Angeles, California, United States

**Keywords:** knockout, ocular infection, latency-reactivation, primary infection

## Abstract

**Purpose:**

T_H_17 cells play an important role in host defense and autoimmunity yet very little is known about the role of IL17 in herpes simplex virus (HSV)-1 infectivity. To better understand the relationship between IL17 and HSV-1 infection, we assessed the relative impact of IL17A-deficiency and deficiency of its receptors on HSV-1 responses in vivo.

**Methods:**

We generated IL17RA^−/−^ and IL17RA^−/−^RC^−/−^ mice in-house and infected them along with IL17A^−/−^ and IL17RC^−/−^ mice in the eyes with 2 × 10^5^ PFU/eye of wild type (WT) HSV-1 strain McKrae. WT C57BL/6 mice were used as control. Virus replication in the eye, survival, corneal scarring (CS), angiogenesis, levels of latency-reactivation, and levels of CD8 and exhaustion markers (PD1, TIM3, LAG3, CTLA4, CD244, and CD39) in the trigeminal ganglia (TG) of infected mice were determined on day 28 postinfection.

**Results:**

No significant differences in virus replication in the eye, survival, latency, reactivation, and exhaustion markers were detected among IL17A^−/−^, IL17RA^−/−^, IL17RC^−/−^, IL17RA^−/−^RC^−/−^, and WT mice. However, mice lacking IL17 had significantly less CS and angiogenesis than WT mice. In addition, angiogenesis levels in the absence of IL17RC and irrespective of the absence of IL17RA were significantly less than in IL17A- or IL17RA-deficient mice.

**Conclusions:**

Our results suggest that the absence of IL17 protects against HSV-1-induced eye disease, but has no role in protecting against virus replication, latency, or reactivation. In addition, our data provide rationale for blocking IL17RC function rather than IL17A or IL17RA function as a key driver of HSV-1-induced eye disease.

Because original reports showed the role of CD4^+^ T cell subsets (T_H_1 and T_H_2) and CD8^+^ T cell subtypes (T_C_1 and T_C_2),^1^^–^^3^ additional types of helper T cells have been identified, including regulatory T cells (T_reg_), type 17 cells (T_H_17), follicular helper T cells (T_fh_), and type 9 cells (T_H_9).^4^^–^^7^ T_H_17 cells play an important role in controlling some pathogens in various infection and autoimmune disease models.^8^ The T_H_17 subset of T cells produces six cytokines (IL17A, IL17B, IL17C, IL17D, IL17E/IL25, and IL17F) and expresses five receptors (IL17RA, IL17RB, IL17RC, IL17RD and IL17RE).^8^^–^^10^ However, the functions of these receptors in relation to the outcome of disease management are not well characterized. IL17A is a major member of the T_H_17 cytokine family and is highly conserved among vertebrates.^11^ It has been implicated in the pathogenesis of many common autoimmune disorders, including multiple sclerosis (MS), rheumatoid arthritis (RA), psoriasis, and inflammatory bowel disease.^8^^,^^12^^–^^19^ Two anti-IL17A monoclonal antibodies were approved for treatment of psoriasis, spondyloarthropathies, psoriatic arthritis, and ankylosing spondylitis,^20^ although both these antibody therapies had significant side effects.

IL-17A is produced by T_H_17 cells that develop along a pathway distinct from T_H_1 and T_H_2 pathways.^21^^,^^22^ T_H_ cell subsets are defined by their signature cytokine and lineage-specific transcription factors.^23^ It is well established that both IL17-producing CD4^+^^24^ and CD8^+^ T cells^25^ play important roles in autoimmunity. In addition to T_H_1 and T_H_2, naive T cells can also differentiate into T_H_17 cells that secrete IL17A^21^^–^^23^^,^^26^ and, in addition to ILCs, CD4^−^, CD8^−^ T cells, γδT cells, invariant natural killer (iNK) T cells, natural killer (NK) cells, neutrophils, and mast cells have been shown to secrete IL17A.^27^^–^^31^ IL17A and IL17F both bind to IL17 receptors A (IL17RA) and C (IL17RC), and this engagement activates mitogen-activated protein kinases (MAPKs), nuclear factor-kappa B (NF-kB), and CCAAT-enhancer-binding protein (C/EBP) signaling pathways through the adaptor proteins Act1 and TRAF6.^32^^,^^33^ Thus, IL17RC is an obligate coreceptor with IL17RA for signaling induced by IL17A and IL17F. IL17RC is also required for IL17A-dependent and IL17F-dependent signaling and has been implicated in the pathogenesis of experimental autoimmune encephalomyelitis (EAE).^34^

Extensive studies have evaluated the role of CD4^+^ and CD8^+^ T cells in herpes simplex virus (HSV)-induced eye disease.^35^^–^^37^ However, little is currently known about the role of T_H_17 in viral infection and viral-induced tissue damage in general, and in HSV-1 infection in particular. Published studies suggest T_H_17 cells protect against certain diseases and infection, and are associated with autoimmunity and pathogenesis.^8^^,^^12^^–^^19^^,^^38^^–^^41^ Previous studies implicating T_H_17 cells in diseases, such as psoriasis, inflammatory bowel disease, RA, EAE, and MS, led us to examine the possibility that IL17A, IL17RA, and IL17RC regulate HSV-1 infectivity in vivo.

The effects of deficiency in IL17A, IL17RA, IL17RC, or in both IL17RA and IL17RC on virus replication in the eye, survival, corneal scarring, angiogenesis, latency, T cell exhaustion, and explant reactivation were determined in HSV-1 ocularly infected mice. Our results, similar to published studies implicating T_H_17 cells in psoriasis, inflammatory bowel disease, RA, EAE, and MS, have shown that T_H_17 cells plays a pathogenic role in eye disease in ocularly infected mice. Our results suggest that T_H_17 has pro-pathogenic and pro-inflammatory functions and may enhance pathology in the eyes.

## Results

### Construction of IL17RA^−/−^ and IL17RA^−/−^RC^−/−^ Mice

**Figure 1.**
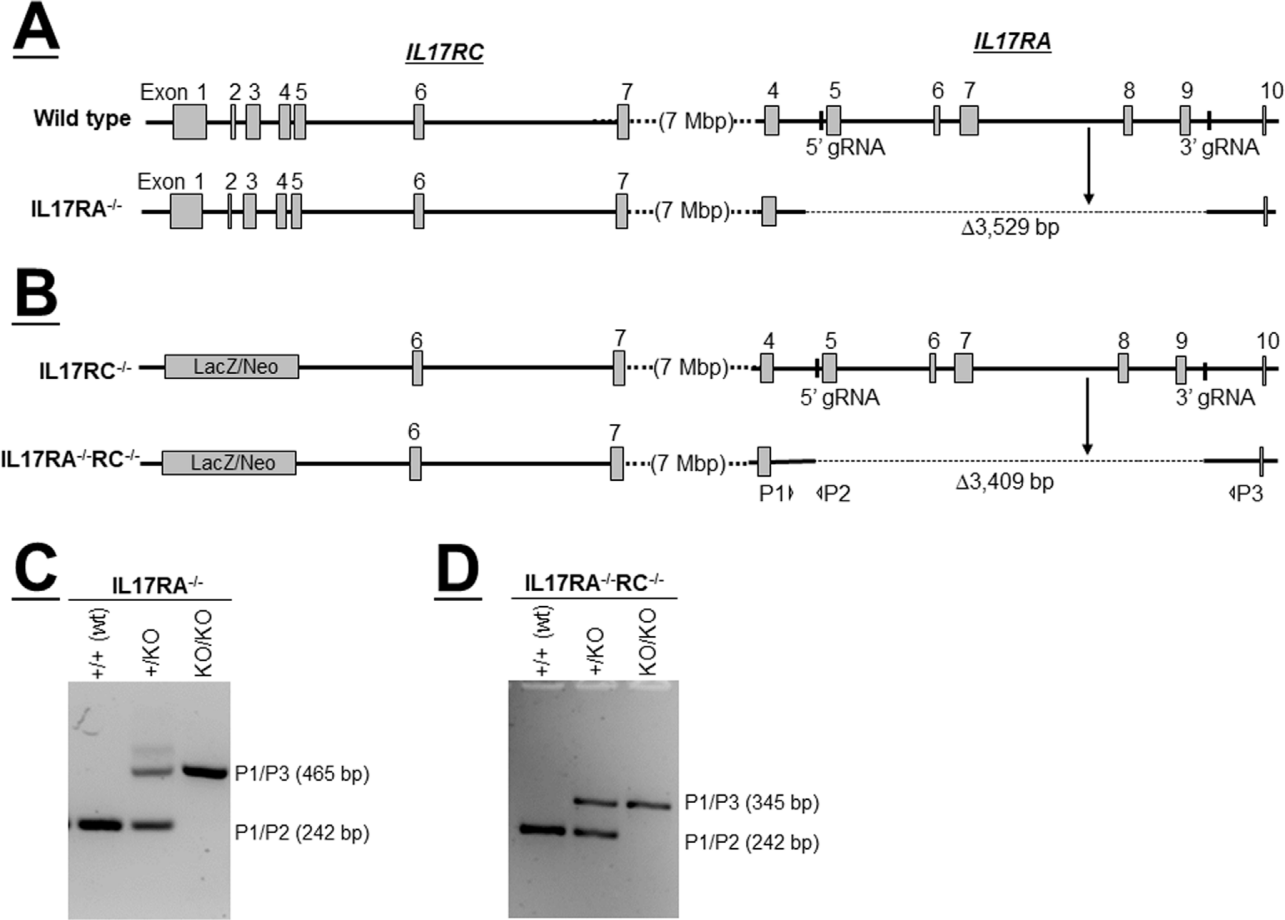
Generation of IL17RA^−/−^ and IL17RA^−/−^RC^−/−^ mice. (A) Schematic diagram of IL17RA^−/−^ mice. Top diagram depicts genomic loci of IL17RC and IL17RA in wt mice. Vertical bars show binding sites of guide RNAs (gRNAs) used to target Cas 9 to exons 5 and 9 of IL17RA using wild type mice (exons are shown as gray boxes). Deletions generated by CRISPR method are shown as dotted lines in the bottom diagram; (B) Schematic diagram of IL17RA^−/−^RC^−/−^ mice. Top diagram shows genomic loci of IL17RC and IL17RA in IL17RC^−/−^ mice. Similar to A above, gRNAs were used to delete exons 5 to 9 of IL17RA in IL17RC^−/−^ mice. Deletions generated by CRISPR method are shown as dotted lines in the bottom diagram, and P1, P2, and P3 indicate binding sites of primers used to detect deletions by PCR; (C) PCR to detect deletion of IL17RA in wt mice. DNA from mice generated in A was used to confirm deletion of IL17RA. PCR primers P1 and P2 yield the PCR product from wild type allele, while PCR primers P1 and P3 yield PCR products from the knockout alleles; and (D) PCR to detect deletion of IL17RA in IL17RC^−/−^ mice. DNA from mice generated in B was used to confirm deletion of IL17RA in IL17RC^−/−^ mice. PCR primers P1 and P2 yield the PCR product from wild type allele, while PCR primers P1 and P3 yield PCR products from the knockout alleles.

To study T_H_17 function in HSV-1 infectivity, IL17A^−/-^ and IL17RC^−/−^ mice were obtained commercially, whereas IL17RA^−/−^ and IL17RA^−/−^RC^−/−^ mice were generated as described in Materials and Methods. IL17RA and IL17RC loci are separated by only 7 mega base pairs on chromosome 6, making them too close to generate IL17RA^−/−^RC^−/−^ mice by crossing IL17RA^−/−^ mice with IL17RC^−/−^ mice. Therefore, we first generated IL17RA^−/−^ mice by disrupting the IL17RA alleles in fertilized eggs of wild type C57BL/6J (JAX 000664) mice using CRISPR genome editing technology as described in Materials and Methods (Fig. 1A). We then generated IL17RA^−/−^RC^−/−^ mice by applying the same method to fertilized eggs of IL17RC^−/−^ mice (Fig. 1B). We verified successful deletion of exons 5 to 9 of IL17RA, which are essential for IL17A binding,^39^ by PCR in wild type (WT; Fig. 1C) and in IL17RC^−/−^ (Fig. 1D) mice.

### Replication of HSV-1 in the Eyes of IL17A- and its Receptor-Deficient Mice

**Figure 2.**
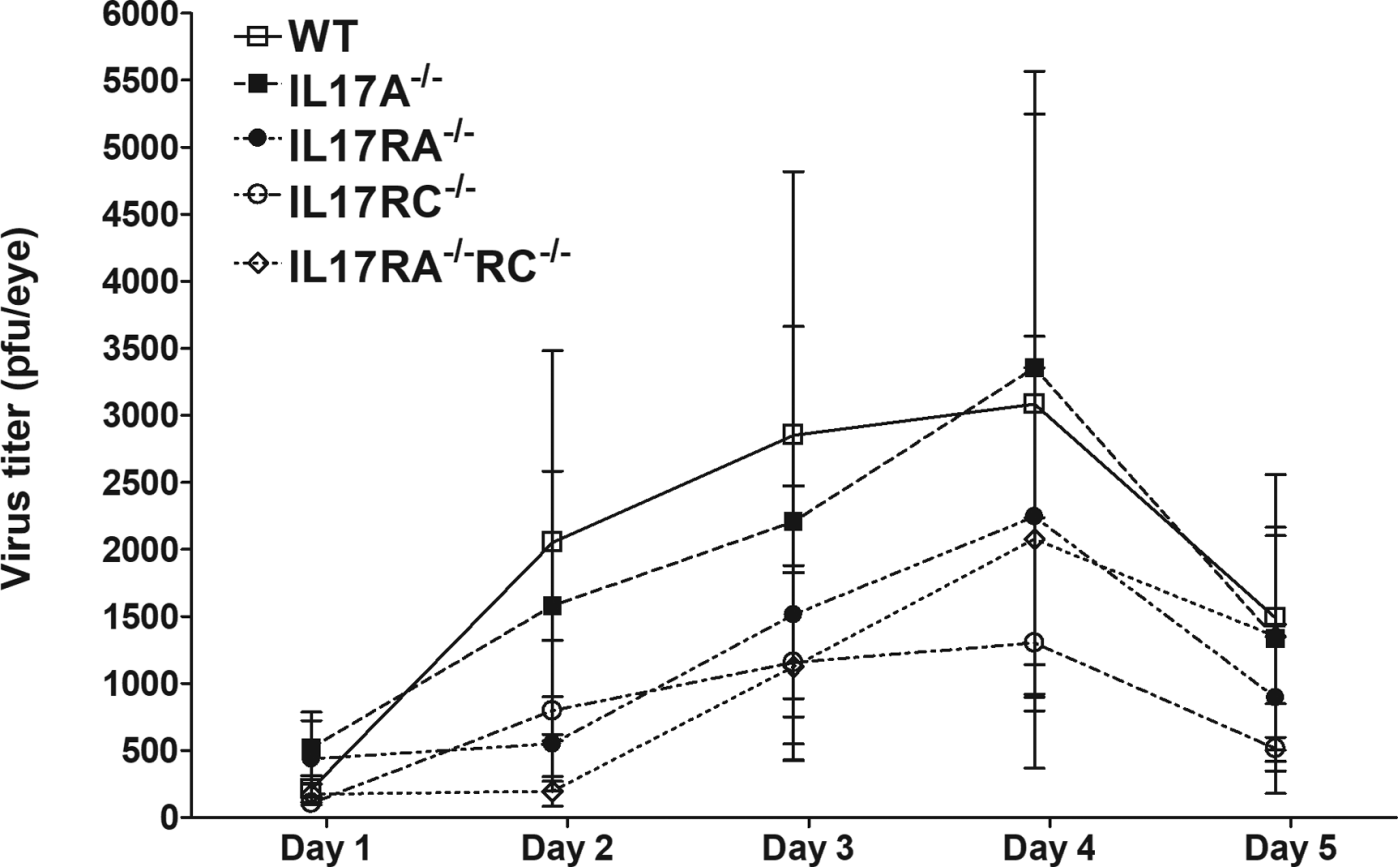
Virus titers in the eyes of infected mice. WT and IL17A^−/−^, IL17RA^−/−^, IL17RC^−/−^, and IL17RA^−/−^RC^−/−^ mice were infected with 2 × 10^5^ PFU/eye of HSV-1 strain McKrae as described in the Materials and Methods. Tear films were collected on days 1-5 PI and virus titers were determined using standard plaque assays. Each point represents the mean ± SEM titers of 28 eyes for WT, 20 eyes for IL17A^−/−^; 26 eyes for IL17RA^−/−^; 26 eyes for IL17RC^−/−^; and 22 eyes for IL17RA^−/−^RC^−/−^ mice from 2-4 separate experiments.

To assess the effects of IL17 deficiency on HSV-1 infection in vivo, IL17A^−/−^, IL17RA^−/−^, IL17RC^−/−^, and IL17RA^−/−^RC^−/−^ mice were infected in the eyes with 2 × 10^5^ PFU/eye of HSV-1 strain McKrae. WT mice were used as a control. Tear films were collected from day 1 to day 5 postinfection (PI) and infectious virus titers in the tear films were determined by standard plaque assays. Virus titers in the eyes were similar among all four groups of knockout mice and WT mice between days 1 and 5 PI (Fig. 2; *P* > 0.05; ANOVA), suggesting that the absence of IL17A or its receptors did not affect HSV-1 replication in the eyes of infected mice.

### Virulence in Infected IL17-deficient Mice

Survival over 4 weeks was monitored in three separate experiments using groups of WT, IL17A^−/−^, IL17RA^−/−^, IL17RC^−/−^, and IL17RA^−/−^RC^−/−^ mice that had been infected in both eyes with 2 × 10^5^ PFU/eye of McKrae. Two mice in the WT (2 of 25) and IL17A^−/−^ (2 of 26) groups died (Table). All of the infected IL17RA^−/−^ mice (29 of 29) survived; 22 of 26 IL17RC^−/−^ mice survived, and one mouse in the IL17RA^−/−^RC^−/−^ (1 of 23) group died. We did not find statistically significant differences in survival between any of the groups of infected deficient mice and WT mice (Table; *P* > 0.5; ANOVA). These results suggest that absence of IL17A or its receptors did not alter survival in the eyes of infected mice.

**Table.** Survival of Knockout Mice Following Ocular Infection^a^

Mouse Strain	Survival/Total
WT	23/25 (92%)
**IL17A^−^^/^^−^**	24/26 (92%)
IL17RA^−/−^	29/29 (100%)
IL17RC^−/−^	22/26 (85%)
IL17RA^−/−^RC^−/−^	22/23 (96%)

a Mice were infected with 2 × 105 PFU/eye of HSV-1 strain McKrae and survival was determined 28 days postinfection as described in Materials and Methods. Survival is from three separate experiments. Differences between different strains of mice were not statistically significant as determined by ANOVA.

### Corneal Scarring and Angiogenesis Decrease in the Eyes of Infected IL17-Deficient Mice

**Figure 3.**
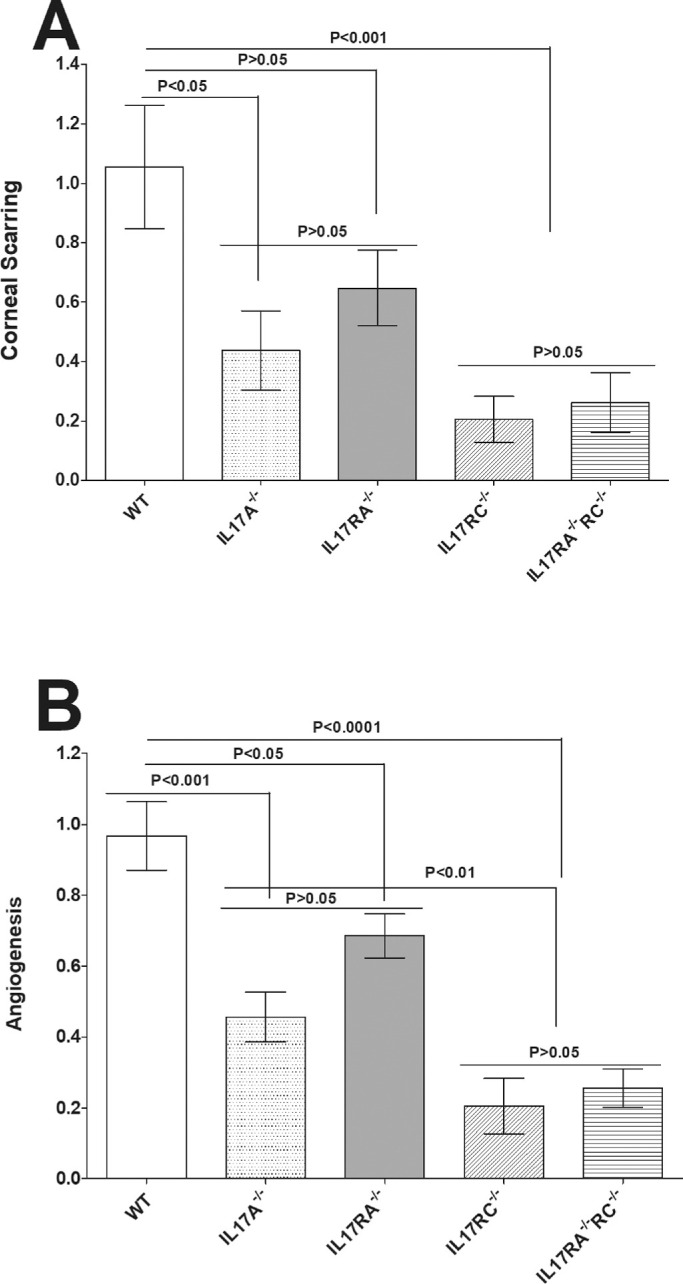
Loss of IL17 contributes to reduced eye disease. Corneal scarring (CS) and angiogenesis in surviving mice were assessed on day 28 PI as described in Materials and Methods. CS and angiogenesis were assessed in 46 eyes from WT, 48 eyes from IL17A^−/−^ mice, 58 eyes from IL17RA^−/−^ mice, 44 eyes from IL17RC^−/−^ mice, and 44 eyes from IL17RA^−/−^RC^−/−^ mice. Experiments were repeated three times and CS and angiogenesis scores are presented as mean ± SEM. p-value was determined using a one-way ANOVA test. Panels: A) CS in surviving mice; and B) Angiogenesis in surviving mice.

To determine the effect of IL17 or its receptors on corneal scarring (CS) and angiogenesis after HSV-1 infection, the eyes of mice that survived ocular infection were examined for CS and angiogenesis on day 28 PI as described in Materials and Methods. The kinetics of eye disease and angiogenesis on day 28 PI are shown in Figure 3. WT infected mice developed significantly more CS than IL17A^−/−^, IL17RA^−/−^, IL17RC^−/−^, and IL17RA^−/−^RC^−/−^ infected mice (Fig. 3A; *P* < 0.0001, 1-way ANOVA), whereas no significant differences in CS were detected among IL17A^−/−^, IL17RA^−/−^, IL17RC^−/−^, and IL17RA^−/−^RC^−/−^ infected mice even though IL17RC^−/−^ and IL17RA^−/−^RC^−/−^ mice had less CS than IL17A^−/−^ and IL17RA^−/−^ mice (Fig. 3A; *P* > 0.05, Tukey's multiple comparison test).

Similar to CS (Fig. 3A), WT mice had significantly more angiogenesis than IL17A^−/−^, IL17RA^−/−^, IL17RC^−/−^, and IL17RA^−/−^RC^−/−^ infected mice (Fig. 3A; *P* < 0.0001, 1-way ANOVA). The level of angiogenesis was not statistically different between IL17A^−/−^ and IL17RA^−/−^ infected mice (Fig. 3B; *P* > 0.05, Tukey's multiple comparison test) or between IL17RC^−/−^ and IL17RA^−/−^RC^−/−^ infected mice (Fig. 3B; *P* > 0.05, Tukey's multiple comparison test). However, the level of angiogenesis was significantly lower in IL17RC^−/−^ and IL17RA^−/−^IL17RC^−/−^ infected mice than in IL17RA^−/−^ infected mice (Fig. 3B; *P* < 0.001, Tukey's multiple comparison test). Similar to other types of autoimmunity, these results suggest that IL17 plays a pathogenic role in the eye. Thus, the absence of IL17 ameliorated CS and angiogenesis induced by ocular infection with HSV-1.

### Reactivation Time is not Affected in the Trigeminal Ganglia of Latently-Infected IL17-Deficient Mice

**Figure 4.**
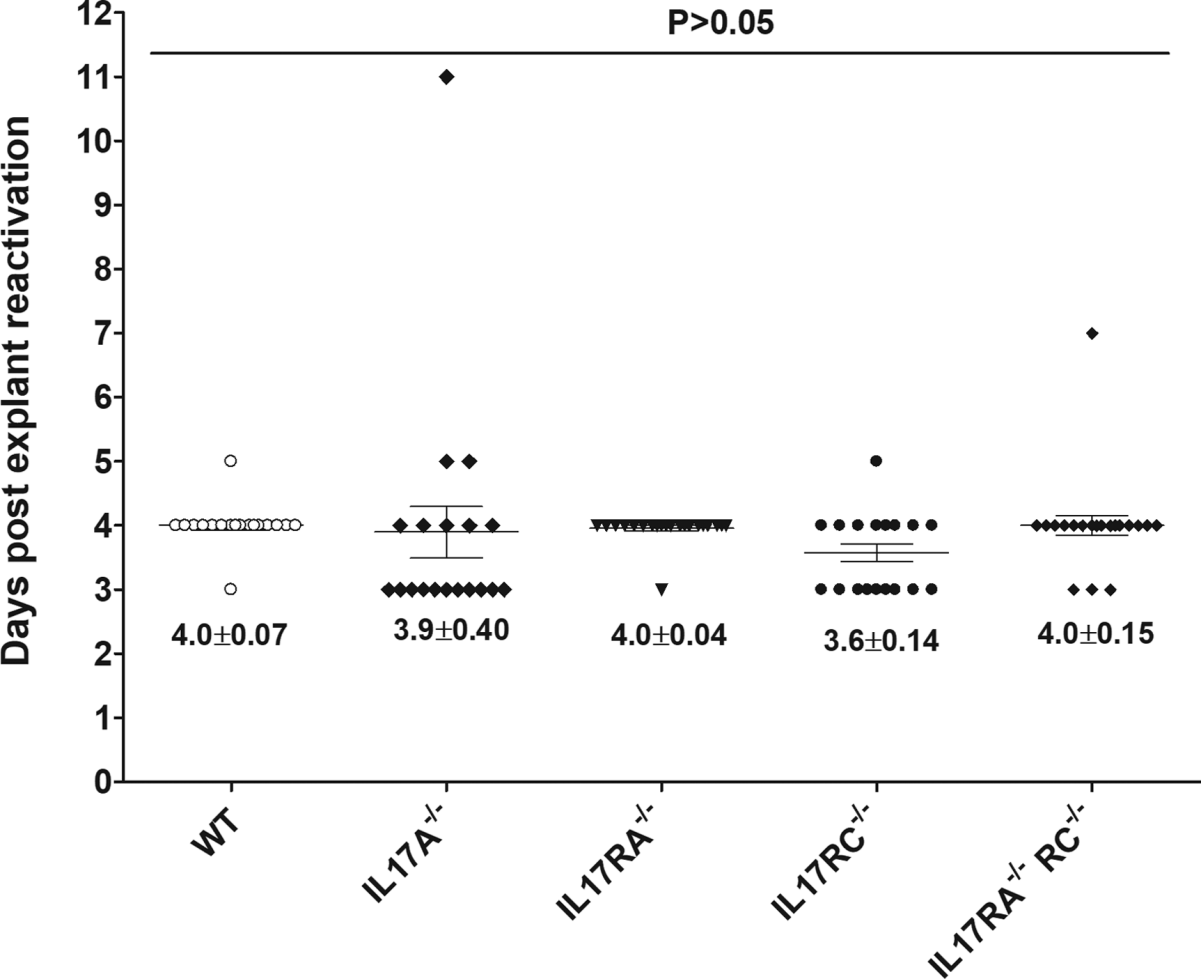
Duration of reactivation is not affected in IL17-deficient mice. To analyze explant reactivation in infected mice, WT and IL17A^−/−^, IL17RA^−/−^, IL17RC^−/−^ and IL17RA^−/−^RC^−/−^ mice were infected in the eye as described in Figure 2. On day 28 PI, TG from infected mice were harvested for explant reactivation. Each individual TG was incubated in 1.5 ml of tissue culture media at 37°C and the presence of infectious virus was monitored for 12 d. Reactivation is based on 21of 22, 20 of 26, 24 of 28,19 of 20, and 23 of 24 TG of WT and IL17A^−/−^, IL17RA^−/−^, IL17RC^−/−^ and IL17RA^−/−^RC^−/−^ mice, respectively. The average time that the TG from each group first showed CPE ± SEM is shown. p-value was determined using a one-way ANOVA test.

Trigeminal ganglia (TG) from some WT and IL17A^−/−^, IL17RA^−/−^, IL17RC^−/−^, and IL17RA^−/−^RC^−/−^ mice that survived ocular infection (Table) were isolated on day 28 PI. Virus reactivation was analyzed using explanted individual TG from infected mice. The times to explant reactivation did not differ significantly between each group of IL17-deficient mice and WT mice or among groups of IL17-deficient mice (Fig. 4; *P* > 0.05, ANOVA). Thus, the absence of IL17A or its receptors did not alter the time to explant reactivation in infected mice, suggesting that T_H_17 cells do not contribute to virus reactivation.

### Latency Levels are not Affected in the TG of Latently-Infected IL17-Deficient Mice

**Figure 5.**
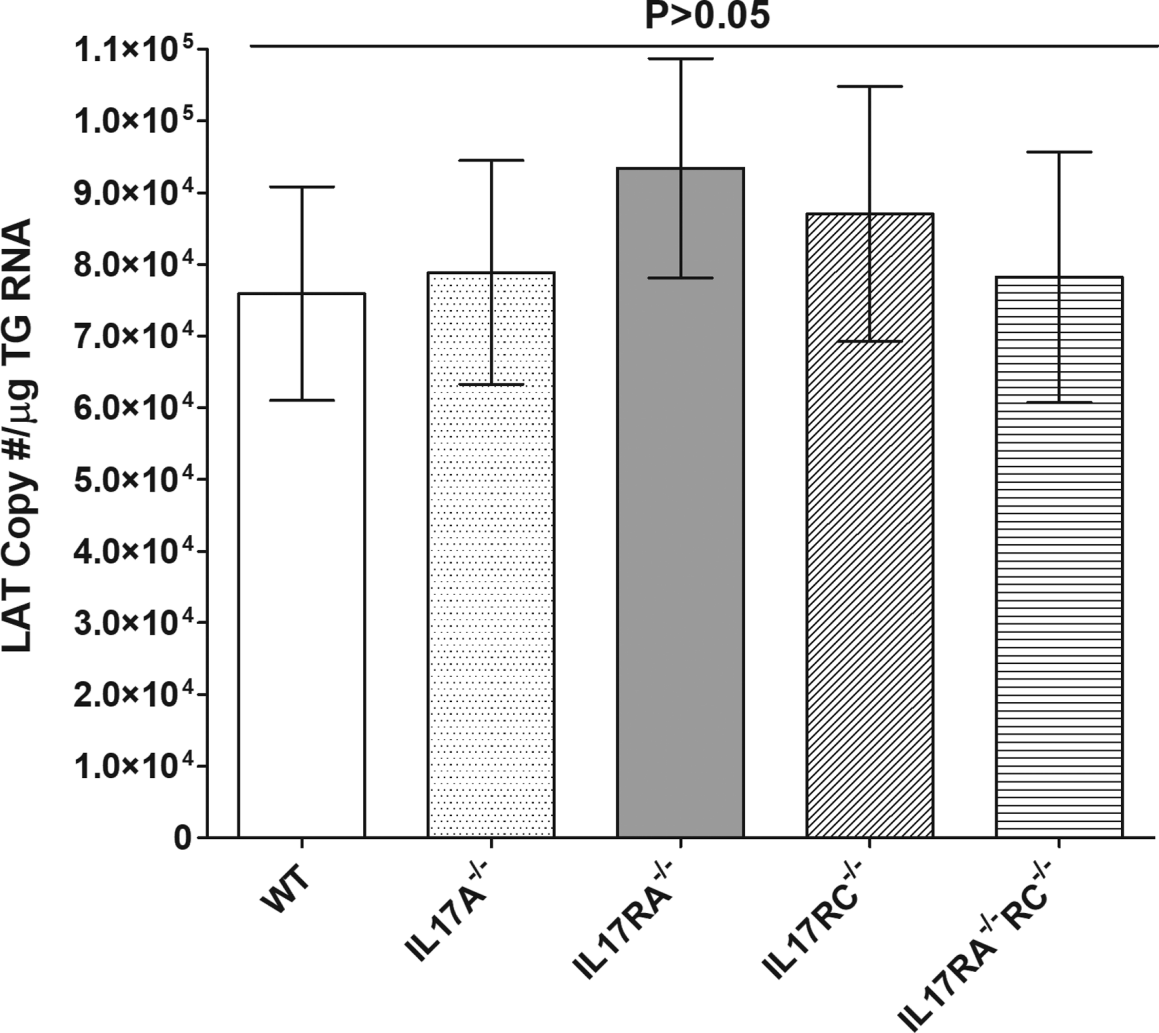
Latency levels in infected mice. To analyze levels of latency in the TG of latently-infected mice, WT and IL17A^−/−^, IL17RA^−/−^, IL17RC^−/−^, and IL17RA^−/−^RC^−/−^ mice were infected in the eye as described in Figure 2 above. On day 28 PI, TG were harvested from the latently-infected mice. Quantitative RT-PCR was performed on individual TG from each mouse. GAPDH expression was used to normalize relative expression of LAT RNA in the TG. LAT copy number per TG were measured using pGEM5317, a LAT containing plasmid as we described previously.^49^ Latency is based on 18 TG per group of mice. p-value was determined using a one-way ANOVA test.

Previously, we have shown that CD8α^−/−^ but not CD8β^−/−^ or β2M^−/−^ mice had less latency than WT mice.^42^^,^^43^ The role of T_H_17 cells in HSV-1 latency is not known. To determine if IL17 modulates latency levels associated with ocular HSV-1 infection, WT, IL17A^−/−^, IL17RA^−/−^, IL17RC^−/−^, and IL17RA^−/−^RC^−/−^ mice were infected with 2 × 10^5^ PFU/eye of HSV-1 strain McKrae. Individual TG from surviving mice were isolated on day 28 PI and total RNA was isolated. LAT RNA levels were quantified using TaqMan RT-PCR. Combined data from two separate experiments showed no significant differences in the amounts of LAT RNA during latency among IL17A^−/−^, IL17RA^−/−^, IL17RC^−/−^, or IL17RA^−/−^RC^−/−^ mice and WT mice (Fig. 5; *P* > 0.5; ANOVA) and LAT RNA levels among IL17A^−/−^, IL17RA^−/−^, IL17RC^−/−^, and IL17RA^−/−^RC^−/−^ mice did not statistically differ (Fig. 5; *P* > 0.2). These results suggest that the absence of IL17 function does not affect latency levels in the TG of mice that have been infected with HSV-1.

### Levels of CD8, PD1, TIM3, LAG3, CD244, CTLA4, and CD39 mRNAs in TG of Latently-Infected Mice

**Figure 6.**
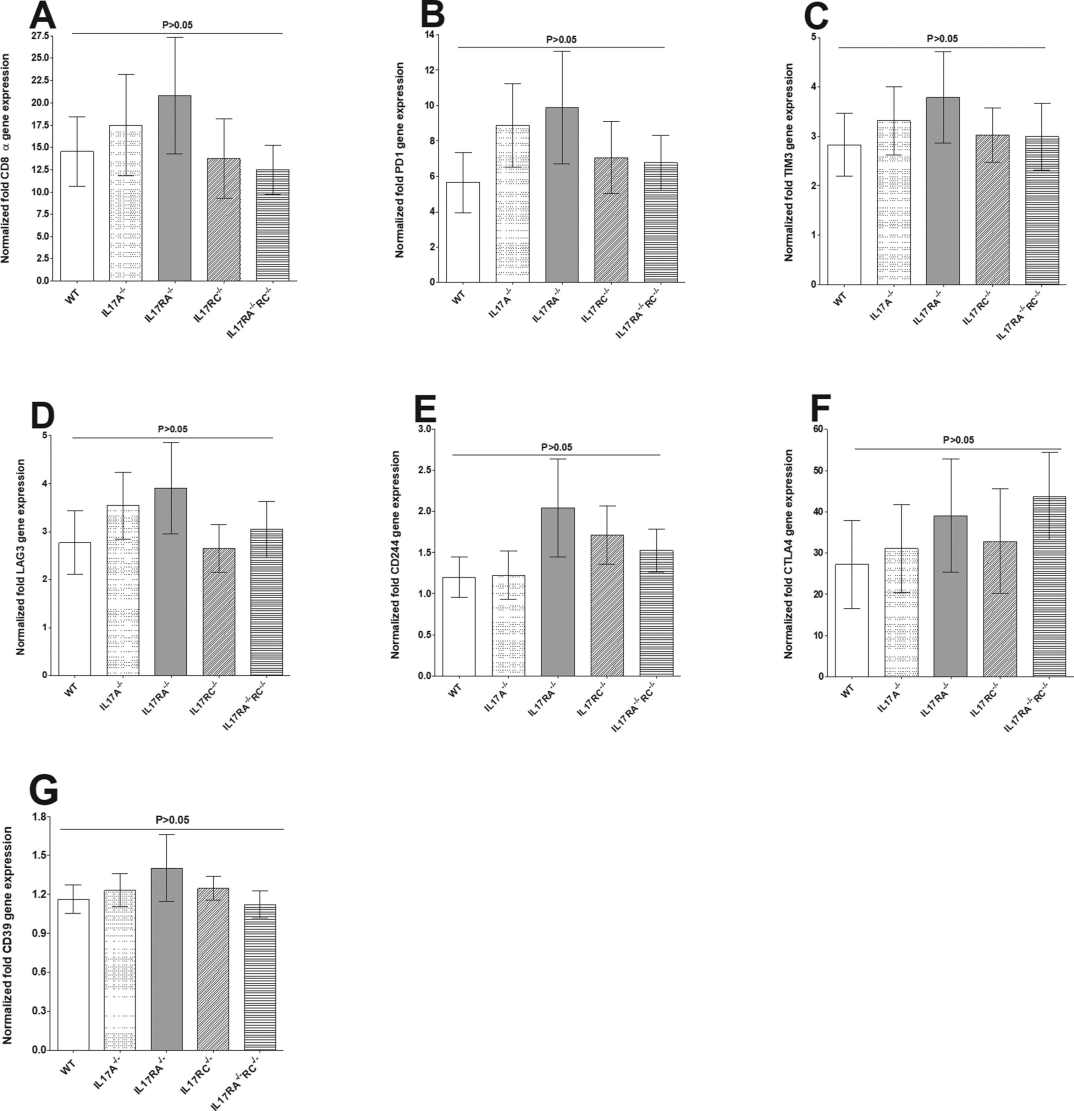
Roles of IL17 on CD8 and exhaustion markers in the TG of latently-infected mice. RNA isolated from WT and IL17A^−/−^, IL17RA^−/−^, IL17RC^−/−^, and IL17RA^−/−^RC^−/−^ mice as described in Figure 5 were used to measure the expression of CD8α, PD1, TIM3, LAG3, CD244, CTLA4, and CD39 in latently-infected TG. qRT-PCR was performed using total RNA as described in the Materials and Methods. CD8α, PD1, TIM3, LAG3, CD244, CTLA4, and CD39 expression in naive WT mice was used as a baseline control to estimate relative expression of each transcript in TG of latently-infected mice. GAPDH expression was used to normalize the relative expression of each transcript. Each point represents the mean ± SEM from 10 to 12 TG for each strain of mice. p-value was determined using a one-way ANOVA test. Panels: A) CD8α transcript; B) PD1 transcript; C) TIM3 transcript; D) LAG3 transcript; E) CD244 transcript; F) CTLA4 transcript; and G) CD39 transcript.

In addition to PD1,^44^ exhausted T cells can express lymphocyte activation gene 3 protein (LAG3),^45^ TIM3 (T cell immunoglobulin and mucin domain–containing protein-3),^46^ CD244 (also known as 2B4),^45^ CTLA4 (cytotoxic T lymphocyte antigen 4),^47^ and CD39 (ectonucleoside triphosphate diphosphohydrolase 1).^48^ Thus, to investigate effects of IL17, absence on T cell exhaustion in the TG of latently infected mice, relative levels of CD8, PD1, TIM3, LAG3, CD244, CTLA4, and CD39 transcripts were determined in the TG of latently infected IL17A^−/−^, IL17RA^−/−^, IL17RC^−/−^, IL17RA^−/−^RC^−/−^, and WT control mice by RT-PCR of total TG RNA extracts. The results are presented in Figure 6 as “fold increase” in infected WT and IL17A^−/−^, IL17RA^−/−^, IL17RC^−/−^, and IL17RA^−/−^RC^−/−^ mice compared to the baseline mRNA levels in the TG from WT uninfected naive mice. Levels of CD8 (Fig. 6A), PD1 (Fig. 6B), TIM3 (Fig. 6C), LAG3 (Fig. 6D), CD244 (Fig. 6E), CTLA4 (Fig. 6F), and CD39 (Fig. 6G) mRNAs in the TG of latently infected IL17A^−/−^, IL17RA^−/−^, IL17RC^−/−^, IL17RA^−/−^RC^−/−^, and WT control mice were higher than the levels in uninfected WT mice. However, we did not find significant differences among CD8, PD1, TIM3, LAG3, CD244, CTLA4, and CD39 mRNA levels between infected IL17-deficient mice and infected WT mice or between the different groups of infected IL17-deficient mice (Fig. 6; *P* > 0.05). These results are consistent with previously observed increases in CD8^+^ T cell numbers and T cell exhaustion markers in the TG of mice latently infected with HSV-1^49^^,^^50^ and further suggest that the absence of IL17 does not affect these parameters.

## Discussion

Viral-induced blindness as a result of ocular HSV-1 infections is a major cause of eye disease in developed countries.^51^^–^^54^ Herpes-induced eye infection results in diseases ranging from blepharitis to conjunctivitis, dendritic keratitis, disciform stromal edema, and necrotizing stromal keratitis.^52^^,^^55^^–^^58^ It is well known that HSV-1-induced CS and, hence, subsequent HSV-1-induced corneal blindness, are caused by an immune response generated by the host against the virus.^35^^,^^36^ Innate and adaptive immune responses have both been shown to play a major role in protecting against ocular HSV-1 infection and disease depending on the infection model.^59^^–^^69^ However, it is universally accepted that CD4^+^ T cells also contribute to HSV-1-induced eye disease in naive mice^36^ and other studies have shown that CD8^+^ T cells enhance eye disease.^35^ The immune response(s) leading to CS following ocular HSV-1 infection is a combination of neutrophils and T-helper responses.^64^^,^^69^ Although both CD4**^+^** T cell-mediated and CD8**^+^** T cell-mediated immune responses have been reported to protect against ocular HSV-1 infection in naive mice,^37^^,^^70^ adoptive transfer and in vivo T cell subset depletion studies suggest that CD8**^+^** T cells alone are sufficient,^71^^–^^74^ CD4**^+^** T cells alone are sufficient,^75^^–^^78^ or that CD8^+^ and CD4^+^ T cells act together^37^^,^^75^^,^^79^ to induce eye disease.

Following ocular HSV-1 infection, both CD4^+^ and CD8^+^ T cell infiltrates contribute to CS and neovascularization.^80^^–^^84^ Specifically, innate and adaptive immune responses have both been implicated in eye disease and many of these cells (i.e. CD4^+^ T cell, CD8^+^ T cell, ILCs, CD4^−^CD8^−^ T cells, γδT cells, iNK T cells, NK cells, neutrophils, and mast cells) secrete IL17A,^21^^–^^31^ which contributes to many autoimmune disorders in humans.^8^^,^^12^^–^^19^ Thus, IL17A secretion by many of the immune cells listed above suggests that the pathology associated with these cell types could be due to IL17A producing cells. IL17A binds to IL17RA and IL17RC receptors to form a heterodimer.^8^^–^^10^

The role of IL17A in HSV-1-induced immune responses is not known. The goal of this study was to determine if IL17-related immune responses are similar to the responses found in other autoimmune disorders of humans and contribute to disease manifestations in the eyes of HSV-1 infected mice. Because it was of interest to examine the role of T_H_17 cells in HSV-1 induced eye disease, we infected IL17A^−/−^, IL17RA^−/−^, IL17RC^−/−^, IL17RA^−/−^RC^−/−^, and WT mice in the eyes with HSV-1 strain McKrae. We did not see any differences among IL17A^−/−^, IL17RA^−/−^, IL17RC^−/−^, IL17RA^−/−^RC^−/−^ mice and WT mice related to virus replication in the eye, survival, latency, reactivation, or T cell exhaustion. In contrast to our results showing that the absence of IL17 did not increase susceptibility of infected mice to ocular HSV-1 infection, it was previously shown that absence of the IL17 pathway increased susceptibility to a variety of extracellular pathogens.^38^^–^^41^

Herpetic blepharitis is an inflammation of the lid margin following ocular HSV-1 infection, and, in the mouse, increased blepharitis usually correlates well with increased HSV-1 replication.^85^ Blepharitis was measured 7 days after ocular challenge as we described previously.^85^ Similar to virus replication in the eye, we did not detect any differences in the level of blepharitis between WT mice and the four strains of knockout mice. These results suggest that IL-17 is not involved in protection against blepharitis. However, in this study, CS and angiogenesis differed significantly among the different IL17-deficient mouse groups when compared to WT mice. Similar to the results of our study, the IL17 pathway has been shown to play a pathogenic role in many human autoimmune diseases, including psoriasis, RA, MS, and inflammatory bowel disease.^8^^,^^12^^–^^19^ Recently, fever induced T_H_17 cells were also shown to induce expression of transcription factors involved in induction of EAE.^86^ IL17 is also known to have a negative effect on the proliferation of hepatitis B virus-related hepatocellular carcinoma.^87^ Further, IL17 has been implicated in different types of human cancers^88^^,^^89^ and may contribute to HBV-associated liver diseases.^90^

Our current study contributes an important observation that the absence of IL17 has a profound effect on minimizing eye disease but has no role in primary or latent infection. Similar to this study, the absence of IL17RA has been shown to reduce virus-induced corneal inflammation.^91^ When compared with IL17A^−/−^ and IL17RA^−/−^ mice, IL17RC^−/−^ and IL17RA^−/−^RC^−/-^ mice had significantly less angiogenesis, and although not statistically significant, lower CS scores. Angiogenesis levels in IL17RC^−/−^ mice were similar to that seen in IL17RA^−/−^RC^−/−^ mice, suggesting that the absence of IL17RC has a more significant effect on reducing angiogenesis than does the absence of IL17RA. This could be due to effects of loss of IL17RC on IL17F signaling, as IL17A and IL17F are both known to use this receptor or could be due to negative feedback mediated by the CBAD subdomain of IL17RA.^92^^,^^93^ Among the six IL17 family cytokines, IL17A and IL17F share approximately 50% homology and can form homodimers as well as heterodimers.^94^^,^^95^ IL17F is also known to produce IL-1β and IL-6 and is thought to have a role in angiogenesis.^96^^,^^97^ Previously, IL-6 was shown to promote corneal inflammation by recruiting neutrophils to the site of HSV-1 infection.^98^ Thus, lower eye disease in IL17RC^−/−^ mice could be associated with the absence of neutrophils, leading to reduced IL-6 expression at the site of infection. Distinct double positive IL17A and IL17F T_H_17 cells induce inflammation in patients with leprosy.^99^ In addition, IL17RC is required for IL17A- and IL17F-dependent signaling.^34^ Moreover, in another study, activation of the IL17F/IL17RC signaling axis worsens pathogen-associated inflammation in lungs.^100^ IL17RC and IL17RA are also shown to be elevated in chronic obstructive pulmonary disease (COPD) in which secreted IL17A stimulates fibroblast growth factor (FGF)-2 and vascular endothelial growth factor (VEGF).^101^ Previously, IL17A has been shown to contribute to increased VEGF expression and promote corneal angiogenesis after ocular HSV-1 infection.^102^ Similar to this study, previously it was shown that IL-17 promotes *pseudomonas aeruginosa* keratitis in the cornea of infected mice.^103^^,^^104^

Our eye disease results indicate that the absence of IL17RC is more effective than the absence of IL17A as the IL17RC receptor targets both IL17A and IL17F secretion. Recently, dual inhibition of both IL17A and IL17F in the treatment of psoriatic disease and ankylosing spondylitis was shown to be more effective than single inhibition of each cytokine.^105^ Other studies have shown that neutralization of both IL17A and IL17F using a human monoclonal antibody suppressed human chronic tissue inflammation.^106^ Thus, reduced angiogenesis in the absence of IL17RC suggests that the absence of IL17RC affects signaling through both IL17A and IL17F, whereas the absence of IL17RA affects IL17A but not IL17F. Taken together, our results and other studies support the hypothesis that IL17F may have a role in HSV-1-induced eye disease and targeting IL17RC may provide better protection and control of eye disease than IL17A.

In summary, our results suggest that the IL17 pathway has a pathogenic role in ocular HSV-1 infection. In addition, similar to other models of autoimmunity, depletion of IL17A and, more importantly, IL17RC may reduce the severity of eye disease after HSV-1 infection by affecting both IL17A and IL17F functions.

## Materials and Methods

### Cells and Virus

Rabbit skin (RS) cells were generated in our laboratory, prepared, grown in MEM media plus 5% FBS and used as we described previously.^59^^,^^107^ Triple plaque-purified virulent HSV-1 strain McKrae was grown in RS cell monolayers as described previously.^108^^,^^109^

### Generation of IL17RA^−/−^ and IL17RA^−/−^RC^−/−^ Mice

A pair of CRISPR guide sequences with the highest specificity scores within introns 4 (386 fwd: ACTTGGTACACAGTGGCGGA followed by a PAM; GGG) and 9 (173 fwd: TTCACTAGCTCTGCACCCGA followed by a PAM; AGG) were selected by using the CRISPOR web algorithm (http://crispor.tefor.net) to cleave exons 5 through 9 that encode fibronectin domains essential for IL17 binding.^110^ The crRNAs and tracrRNA (Cat # U-002000-120) were synthesized by Dharmacon, Inc. (Lafayette, CO). A CRISPR mixture containing 20 ng/µl crRNAs/tracrRNA mix (approximately 1:1 molar ratio) and 20 ng/µl eSPCas9 protein (Cat # ESPCAS9PRO-50UG) (Millipore-Sigma, Burlington*,* MA, USA) in injection buffer (0.1 mM EDTA, 10 mM Tris-HCl, 100 mM NaCl) was introduced into WT C57BL/6J or IL17RC^−/−^ fertilized eggs by pronuclear microinjection via a standard method.^111^ Guide RNA efficacy and status of non-homologous end joining (NHEJ) was validated by PCR genotyping of blastocysts in initial phase and subsequently on tissue samples isolated from transgenic founders and progeny. Briefly, single blastocysts or toe tissue samples were processed and amplified using the KAPA genotyping kit (Cat # KK7352, KAPA Bioscience, Wilmington, MA, USA) with PCR primers P1 (5’- CATTCTCGAGAGTGTGTGCG -3’) and P2 (5’- CCCCTGTCTGATCTGCATGT -3’) flanking the targeted cleavage sites. Deletion of the IL17RA allele was detected with a combination of primers P1 and P3 (5’- CTGAGGAAGAGAGGC AATGG -3’). PCR fragments were isolated from a preparative gel and purified with QIAquick PCR purification kit (Qiagen, Hilden, Germany). The status of introduced mutation(s) was further confirmed by sequencing the PCR fragments (Genewiz, South Plainfield, NJ, USA). Two recombinant founders out of 6 mice produced in the IL17RC^−/−^ background and 4 recombinant founders were confirmed out of 17 mice produced in the WT C57BL/6J background with heterozygous or mosaic mutations with various deletion sizes. Among two recombinant founder mice of IL17RA^−/−^RC^−/−^, founder #15 had one type of deletion (3401 bp). Founder #14 had two different types of deletions (3445 and 3529 bp), and probably was a mosaic of two different clones. Four founder mice of IL17RA^−/−^, #19, 20, 23, and 27 had one type of deletion in each animal and the same deletion was present in founders #19 and #27 (3409 bp), and #20 and #23 (3402 bp). We established a line after two generations of backcrossing with WT C57BL/6J mice to dilute out possible off-target mutations and mosaicism and we chose founder lines #176 and #166 for further colony expansion of IL17RA^−/−^RC^−/−^ and IL17RA^−/−^ mice, respectively. All the mutant mice generated looked heathy and normal, so mutant mice with the largest deletion were used to establish the lines.

### Mice

Inbred C57BL/6J mice were obtained from the Jackson Laboratory (Bar Harbor, ME, USA). IL17A^−/−^ mice were described previously.^39^ IL17RC^−/−^ mice were developed by Genentech^112^ and were obtained from the Mutant Mouse Regional Resource Center (University of California, Davis, CA, USA). All mice used in this study have a B6 background and were bred in-house. Both male and female (6 to 8-week-old) mice were used in the study. All animal procedures were performed in strict accordance with the Association for Research in Vision and Ophthalmology Statement for the Use of Animals in Ophthalmic and Vision Research and the NIH *Guide for the Care and Use of Laboratory Animals* (ISBN 0-309-05377-3). The animal research protocol was approved by the Institutional Animal Care and Use Committee of Cedars-Sinai Medical Center (Protocol 6134).

### Ocular Infection

IL17A^−/−^, IL17RA^−/−^, IL17RC^−/−^, IL17RA^−/−^RC^−/−^, and the WT control mice were infected with 2 × 10^5^ PFU of HSV-1 strain McKrae per eye, in 2 µl of tissue culture media as an eye drop without corneal scarification as we have described previously.^113^

### Titration of Virus in Tears

Tear films were collected from both eyes of infected mice on days 1-5 PI using a Dacron tipped swab. Each swab was placed in tissue culture medium (1 mL) and the amount of virus in the medium was determined using a standard plaque assay on RS cells.^85^

### Monitoring Corneal Scarring in Infected Mice

The severity of CS lesions in the corneas of mice was examined by slit-lamp biomicroscopy. Scoring was as follows: 0, normal cornea; 1, mild haze; 2, moderate opacity; 3, severe corneal opacity but iris visible; 4, opaque and cornea ulcer; and 5, corneal rupture and necrotizing keratitis. The severity of angiogenesis was recorded as we described.^114^

### Monitoring Angiogenesis in Infected Mice

The severity of angiogenesis was recorded by using a system in which a grade of 4 for a given quadrant of the circle represents a centripetal growth of 1.5 mm toward the corneal center. The score of the four quadrants of the eye was summed to derive the neovessel index (range, 0–16) for each eye at a given time point as we described previously.^84^^,^^115^

### In Vitro Explant Reactivation Assay

Mice were euthanized at day 28 PI and individual TG were removed and cultured in 1.5 mL tissue culture medium, as we described previously.^116^ Briefly, a 100 µl aliquot was removed from each culture daily for day 15 and used to infect RS cell monolayers. RS cells were monitored daily for the appearance of cytopathic effect (CPE) for 5 days to determine the time of first appearance of reactivated virus from each TG. As the media from the explanted TG cultures were plated daily, the time at which virus reactivation first occurred in the explanted TG cultures was determined.

### RNA Extraction, cDNA Synthesis, and TaqMan RT-PCR

TG were collected from naive mice and mice that survived ocular infection on day 28 PI and the individual TG were immersed in RNAlater RNA stabilization reagent and stored at -80°C until processing. Tissue processing, total RNA extraction, and RNA yield were carried out as we have described previously.^80^^,^^117^ Following RNA extraction, 1000 ng of total RNA was reverse-transcribed using random hexamer primers and Murine Leukemia Virus (MuLV) Reverse Transcriptase from the High Capacity cDNA Reverse Transcription Kit (ThermoFisher Scientific, Waltham, MA, USA) according to the manufacturer's recommendations. RNA levels were determined using commercially available TaqMan Gene Expression Assays (ThermoFisher Scientific) with optimized primer and probe concentrations. Primer-probe sets consisted of two unlabeled PCR primers and the FAM dye-labeled TaqMan MGB probe formulated into a single mixture. Additionally, all cellular amplicons included an intron-exon junction to eliminate signal from genomic DNA contamination. The following assays were used in this study: (1) CD8α, ABI assay I.D. Mn01182108_m1 – Amplicon length = 68 bp; (2) PD1 (programmed death 1) ABI Mm00435532_m1 – Amplicon size 65 bp; (3) TIM3 (Havcr2 – hepatitis A virus cellular receptor 2) ABI Mm00454540_m1 – Amplicon size 98 bp; (4) CTLA4 (cytotoxic T-lymphocyte-associated protein 4) ABI Mm00486849_m1 – Amplicon size 71 bp; (5) LAG3 (lymphocyte activation gene 3) ABI Mm00493071_m1 – Amplicon size 113 bp; (6) CD244 (CD244 molecule A) ABI Mm00479575_m1 – Amplicon size 148 bp; (7) CD39 (ectonucleoside triphosphate diphosphohydrolase 1) ABI Mm00515447_m1 – Amplicon size 93 bp; and (8) GAPDH was used to normalize transcripts, ABI Mm999999.15_G1 – amplicon length = 107 bp.

The custom-made primers and probe set for LAT were: forward primer, 5′-GGGTGGGCTCGTGTTACAG-3′; reverse primer, 5′-GGACGGGTAAGTAACAGAGTCTCTA-3′; and probe, 5′- FAM-ACACCAGCCCGTTCTTT-3′– Amplicon Length = 81 bp, corresponding to LAT nts 119553-119634. In each experiment, an estimated relative copy number of LAT was calculated using standard curves generated from pGem-LAT5317. Briefly, plasmid DNA template was serially diluted 10-fold such that 5 µl contained from 10^3^ to 10^11^ copies of the desired gene and then subjected to TaqMan PCR with the same set of primers as the test samples. By comparing the normalized threshold cycle of each sample to the threshold cycle of the standards, the copy number for each reaction was determined.

Quantitative real-time RT-PCR (qRT-PCR) was performed using QuantStudio 5 System (Applied Biosystems, Foster City, CA, USA) in 384-well plates as we described previously.^118^^,^^119^

### Statistical Analyses

Student's *t*-test and ANOVA were performed using the computer program Prism (GraphPad, San Diego, CA, USA). Results were considered statistically significant when the *P* value was < 0.05.
